# Dichloroacetate Stabilizes Mitochondrial Fusion Dynamics in Models of Neurodegeneration

**DOI:** 10.3389/fnmol.2019.00219

**Published:** 2019-09-18

**Authors:** Darren O’Hara, Gavin M. Davis, Natalie A. Adlesic, Jerrard M. Hayes, Gavin P. Davey

**Affiliations:** School of Biochemistry and Immunology, Trinity Biomedical Sciences Institute, Trinity College Dublin, Dublin 2, Ireland

**Keywords:** mitochondria, fusion-fission dynamics, carnitine palmitoyl transferase, mitochondrial pyruvate carrier, pyruvate dehydrogenase kinase, Parkinson’s disease, MPP^+^

## Abstract

Mitochondrial dysfunction is a recognized hallmark of neurodegenerative diseases and abnormal mitochondrial fusion-fission dynamics have been implicated in the pathogenesis of neurodegenerative disorders. This study characterizes the effects of metabolic flux inhibitors and activators on mitochondrial fusion dynamics in the neuronal cell culture model of differentiated PC12 cells. Using a real time confocal microscopy assay, it was found that the carnitine palmitoyltransferase I (CPTI) inhibitor, etomoxir, reduced mitochondrial fusion dynamics in a time-dependent manner. Etomoxir also decreased JO_2_, ΔΨ_m_ and reactive oxygen species (ROS) production rates. The mitochondrial pyruvate carrier (MPC) inhibitor, UK5099, reduced fusion dynamics and in combination with etomoxir these inhibitory effects were amplified. Use of the pyruvate dehydrogenase (PDH) kinase inhibitor dichloroacetate, which is known to increase metabolic flux through PDH, reversed the etomoxir-induced effects on fusion dynamics, JO_2_, ΔΨ_m_ but not ROS production rates. Dichloroacetate also partially reversed inhibition of mitochondrial fusion dynamics caused by the parkinsonian-inducing neurotoxin, MPP^+^. These results suggest that dichloroacetate-induced activation of metabolic flux in the mitochondrion may be a mechanism to restore normal mitochondrial fusion-fission dynamics in metabolically challenged cells.

## Introduction

An imbalance of mitochondrial fusion-fission dynamics is present in models of neurodegeneration and Parkinson’s disease (PD; Knott and Bossy-Wetzel, 2008; Poole et al., 2008; Yang et al., 2008; Büeler, 2009; Kamp et al., 2010; Santos et al., 2015). Highly fused mitochondrial networks are generated in cell types when increased respiratory capacity and bioenergetics function is required (Westermann, 2012). Nutrient excess increases respiration rates and a robust fragmentation of the mitochondrial network (Molina et al., 2009), while low availability of nutrient sources elongates mitochondria by inhibiting recruitment of fission-inducing Drp1 to the mitochondria (Gomes et al., 2011).

Neurons primarily use glucose as the main carbohydrate for catabolic metabolism but under certain conditions can utilize fatty acids as energy substrates. During starvation, fatty acids are a source of cellular energy and fatty acid import into mitochondria is sourced directly by lipid droplets (LDs) in close proximity to highly fused mitochondria (Rambold et al., 2015). When mitochondrial fusion is inhibited in starved cells, fatty acids are not efficiently metabolized and re-associated with LDs and flux into neighboring cells. This suggests a direct link between fatty acid oxidation and mitochondrial fusion, as stimulation of fusion is required to utilize fatty acids as an energy source.

Transport of fatty acids into the mitochondria for β-oxidation is mediated by a carnitine carrier system. Carnitine palmitoyltransferase I (CPT1) is the main rate-limiting step in β-oxidation and represents a key regulatory site controlling flux through this pathway by virtue of its inhibition by the fatty acid synthesis precursor malonyl-CoA (McGarry and Mannaerts, 1977). CPTs are important for astrocytic and neuronal function in the brain (Jernberg et al., 2017; Kuter et al., 2019). The CPT1c isoform is expressed only in neurons (Sierra et al., 2008; Lee and Wolfgang, 2012) and palmitoyl-CoA is a recognized substrate for CPT1c in the PC12 neuronal cell culture model (Sierra et al., 2008).

Neurons are reliant on pyruvate produced during glycolysis due to the apparent low activity of enzymes involved in β-oxidation in neurons (Panov et al., 2014). Pyruvate transported into the mitochondria, facilitated by the mitochondrial pyruvate carrier (MPC), is converted by pyruvate dehydrogenase (PDH) to acetyl CoA which enters the TCA cycle. The MPC regulates energy metabolism and attenuates neurodegeneration (Quansah et al., 2018) and autophagy, inflammation and neurodegeneration in experimental models of PD (Ghosh et al., 2016).

Metabolic flux through PDH is negatively regulated by PDH kinase (PDHK) activity. In neurons, reduced PDH activity is present in several neurological diseases (Sorbi et al., 1983). Reduced levels of PDH activity are present in Lewy body disease and PARK14 familial PD (Miki et al., 2017). Regulation of PDH activity by PDHK has been found in experimental models of PD (Klivenyi et al., 2004; Requejo-Aguilar et al., 2014; Shi and McQuibban, 2017). The PDHK inhibitor dichloroacetate is known to affect glucose and lactate metabolism in the brain (Itoh et al., 2003). Dichloroacetate also improves mitochondrial function in ALS models (Miquel et al., 2012), attenuates neuronal damage in ischemia (Dimlich and Marangos, 1994) and improves survival and neurologic outcomes in rats after cardiac arrest (Wang et al., 2018).

The above evidence suggests that fatty acid and pyruvate metabolism may affect mitochondrial function in the brain and that subsequent dysregulation of mitochondrial fusion-fission dynamics may be important for neurodegenerative processes. Therefore, this study investigates the effects of etomoxir (CPT1 inhibitor), phenylpyruvate (acetylCoA carboxylase activity inhibitor), UK5099 (mitochondrial pyruvate transporter inhibitor) and dichloroacetate (PDHK activity inhibitor) on mitochondrial fusion dynamics in differentiated PC12 cells. In addition, the effects of the parkinsonian-inducing neurotoxin MPP^+^, a specific inhibitor of the mitochondrial electron transport chain complex I activity, on fusion dynamics is tested. The potential for altering metabolic flux in mitochondria with dichloroacetate to restore normal mitochondrial dynamics following metabolic control point inhibition is also considered.

## Materials and Methods

### Cell Culture and DNA Transfection

The rat pheochromocytoma cell line, PC12 (6–15), was cultured in Nunc cell culture treated EasYFlasks (Fisher Scientific, Leicestershire, UK) in RPMI 1640-GlutaMAX (Thermo Fisher) containing 10% (v/v) fetal bovine serum (Sigma) and penicillin-streptomycin solution (100 units/ml penicillin G, 0.1 mg/ml streptomycin sulfate; Sigma) at 37°C in a humidified atmosphere of 5% CO_2_. For differentiation, cell culture flasks and dishes were coated with a solution of 40 μg/ml poly-D-lysine hydrobromide (Sigma) for 2 h and cells were maintained in RPMI-1640-GlutaMAX containing 1% (v/v) fetal bovine serum, penicillin-streptomycin solution and 100 ng ml^−1^ nerve growth factor-2.5S from murine submaxillary gland (Sigma) for 48 h at 37°C in a humidified atmosphere of 5% CO_2_. For transient transfection, cells were seeded in uncoated six well dishes 24 h prior to transfection at a density of 2.5 × 10^5^ cells/well. The following day 3 μg of DNA (pDsRed2-mito and PA-GFP-mito) was diluted in 250 μl of Opti-MEM^TM^ and mixed with 6 μl of Lipofectamine 2000 pre-diluted in 250 μl of the same medium for each well. This mixture was incubated at room temperature for 20 min to allow DNA-Lipofectamine 2000 complexes to form and 500 μl of mixture was added to each well. The plate was shaken gently, and cells were incubated at 37°C in a 5% CO_2_ incubator for 24 h before medium was removed, cells were trypsinized, seeded in poly-d-lysine coated μ-dishes (Ibidi) and differentiated. Differentiated cells were used for live cell confocal microscopy 48 h post-differentiation.

### Generation of Stable Cell Lines

PC12 (6–15) cells were transfected using Lipofectamine according to manufacturer’s instructions. Briefly, cells were seeded in 6-well plates at 2 × 10^5^ cells/well and used once 80% confluent. Two-hundred and fifty microliter Opti-MEM^TM^ solution was mixed with 6 μl Lipofectamine and incubated for 5 min. In another Eppendorf tube 3 μg A53T α-syn plasmid was added to 250 μl Opti-MEM^TM^ and was mixed with the Lipofectamine/Opti-MEM^TM^ mixture. The mixture was left to incubate for 20 min, transfection media (10% FBS, no Penicillion Streptomycin) was added to the cells and the mixture was then added as well and left for 4 h. The media was then replaced with normal media and left for 48 h before cells were seeded at 1,000 cells/well in a 96-well plate with the addition of selection antibiotics. Wells containing positively transfected cells growing from one single colony were chosen, grown up and screened for α-syn overexpression.

### Cell Viability

AlamarBlue^®^, containing the active ingredient resazurin, was used to measure cell viability. PC12 (6–15) cells were seeded in 96-well plates coated with poly-D-lysine at a density of 30,000 cells/well and differentiated for 48 h. AlamarBlue^®^ was added to each well (10%) and incubated for 4 h before fluorescence was measured at excitation *λ* = 570 nm and emission *λ* = 600 nm. Inhibitors were added 1 h prior to reading of the plate.

### ROS Measurement

The dye 2′,7′-dichlorodihydrofluorescein di-acetate (H_2_DCFDA) was used to measure reactive oxygen species (ROS) production in differentiated PC12 cells. Cells were seeded in black 96-well plates with clear bottom coated with poly-D-lysine at a density of 30,000 cells/well and differentiated for 48 h. Cells were washed with warm PBS, before Krebs buffer containing 5 μM H_2_DCFDA was added to each well. Fluorescence was read over 1 h at excitation *λ* = 485 nm and emission *λ* = 530 nm and the initial rate was calculated by getting the rate of increase in fluorescence over the first 15 min. Inhibitors were added 1 h prior to addition of H_2_DCFDA and maintained throughout the measurement.

### Quantification of ΔΨ_m_

ΔΨ_m_ was measured using the ΔΨ_m_-dependent dye tetramethylrhodamine, methyl ester (TMRM). Cells were seeded in poly-D-lysine coated confocal dishes at a density of 80,000 cells/quadrant and differentiated for 48 h. Cells were then loaded with 20 nM TMRM for 30 min before being washed with Krebs buffer and incubated in Krebs buffer containing 5 nM TMRM for the duration of imaging. Excitation with 543 nm laser set to 1.4% power output allowed for the visualization of red-emitting TMRM within mitochondria of cells, viewed through a 63× oil objective. Five randomly chosen fields were pre-selected using the software’s “Mark and Find” facility and imaged. Inhibitors were added 1 h prior to the commencement of imaging and maintained throughout. Using Imaris software, the mean pixel intensity of all red fluorescence in a field of view was calculated in control and treated cells.

### Oxygen Respiration Measurements

A seahorse extracellular flux analyzer XF24 was used for measurement of extracellular acidification rate (ECAR) and oxygen consumption rate (OCR). These measurements were used as an indicator of glycolysis and oxidative phosphorylation rates respectively. PC12 (6–15) cells were seeded at a density of 80,000 cells/well and differentiated for 48 h in a poly-D-lysine coated 24-well cell culture plate. Eighteen hours prior to the beginning of the experiment, a seahorse cartridge was hydrated using calibration buffer in a -CO_2_ incubator at 37°C. On the day of the experiment, seahorse media was supplemented with 10 mM glucose, 2 mM l-glutamine and NGF and warmed to 37°C. Cells were incubated in seahorse media and the plate was placed in a -CO_2_ incubator for 45 min prior to the initiation of the experiment. During this incubation, inhibitors (oligomycin, FCCP, rotenone/antimycin A and 2-deoxyglucose) in seahorse media were added to designated ports of the seahorse cartridge and the plate was then calibrated in the analyzer.

### Live Cell Confocal Microscopy

Live cells were imaged using an Olympus FV1000 point scanning confocal microscope, FV10-ASW Olympus fluoview Ver.2 software and a 60× oil immersion objective and later using a Leica SP8 gated STED microscope, Leica Application Suite X software and a 60× oil immersion microscope. Sequential excitation at 405 nm, 488 nm and 546 nm were provided by near-violet laser diode, argon and helium neon gas lasers, respectively. The imaging chamber on both microscopes was heated to 37°C and had a humidified atmosphere of 5% CO_2_. The method of quantification of mitochondrial fusion rates was based on that of Karbowski et al. (2004), with modifications, and relied on the principle that mitochondria share matrix contents upon fusion. By conferring high fluorescence to a small group of mitochondria within the cellular mitochondrial matrix (photoconverting mitochondria-expressed GFP to a state of ~20-fold increased fluorescence in a small region of interest, or ROI), mitochondrial fusion can be monitored by measuring the rate of decrease in fluorescence intensity in the ROI as matrix contents are shared by fusing organelles. All experiments were carried out using the Olympus FV1000 point scanning Confocal Microscope, FV10-ASW Olympus Fluoview Ver. 2 software and a 60× oil immersion objective with 2× zoom applied or the Leica TCS SP8 confocal microscope, Leica SP8 software and a 60× oil immersion objective set with 2× zoom applied. Sequential excitation at 488 nm and 542 nm allowed for visualization of PA-GFP and DsRed protein expression in transfected cells respectively. Using the software’s Multiple Time Lapse facility, five separate fields were pre-selected per group (control and treated) per dish, each containing at least one cell. Cells were imaged with z-stacking (6–7 slices of 0.8–1.4 μm thickness) before and after photoactivation of discrete 6.25 μm^2^ regions of interest (ROIs) of the mitochondrial network, with post-activation imaging intervals set at 1, 15, 30 and 45 min. Using the software’s Image Analysis facility, the mean pixel intensities of the photoactivated ROIs were calculated in both the red and green channels at all time points and expressed as a percentage of pixel intensity 1 min post-activation, deemed to be the point at which pixel intensity was highest.

### Statistics

Statistical analyses were performed with Prism 6. Data are obtained from at least three independent experiments and experiments and expressed as mean ± SEM unless otherwise specified. The Student’s *t*-test (unpaired, two-tailed) for parametric data was used for analysis of two groups. In experiments with more than two groups, analysis of variance (ANOVA) was performed. All statistical significance was calculated at *P* = 0.05.

## Results

### Carnitine Palmitoyltransferase and Mitochondrial Pyruvate Carrier Inhibitors Attenuate Mitochondrial Fusion Dynamics

**Figure 1:**
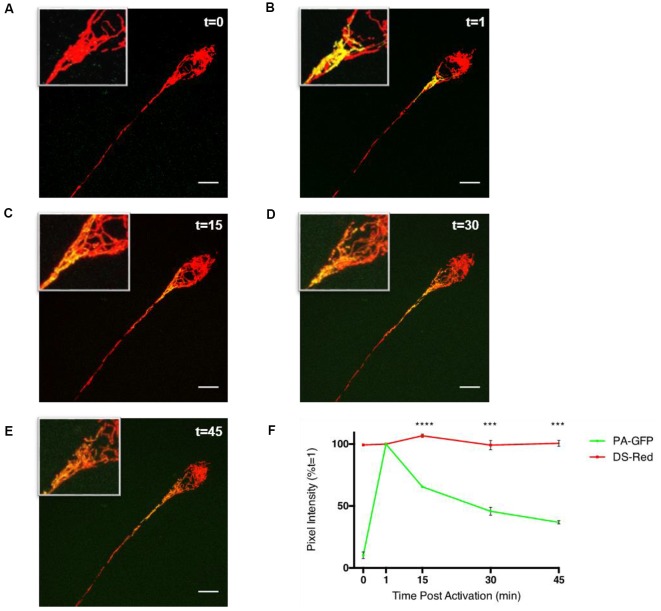
Assay of mitochondrial fusion dynamics in differentiated PC12 cells. Differentiated PC12 cells were transfected with DS-Red and PA-GFP and analyzed by confocal microscopy using a Leica Sp8 microscope. **(A–F)** Photo-activated mitochondria of the cell soma show gradual sharing of fluorescent contents over the duration of a 45 min experiment. Mean pixel intensities of 6.25 μm^2^ photo-activated regions of interest (ROIs) were calculated, initially using Leica SP8 LAS X software, and expressed as a percentage of pixel intensity at 1-min post-activation (*t* = 1) which was the point at which pixel intensity is highest. In the PA-GFP channel, there is a low level of fluorescence prior to activation with a mean pixel intensity of >10% of that of its 1-min post-activation value. The value consistently reduces until it reaches ~40% of its *t* = 1 level 45 min after photo-activation in both cell types. Throughout the experiment, the DS-Red channel showed statistically insignificant fluctuations in pixel intensity at the time points shown indicating the decrease in pixel intensity from the PA-GFP channel were not due to the movement of mitochondria out of the ROI or photobleaching. Data is presented ±SEM, *n* = 3, where five fields, each containing at least 1 cell were imaged in three separate dishes on three separate occasions. Scale bar = 10 μm. Data presented as mean ± SEM, *n* = 3. Unpaired *t*-test at each time point with Welch correction for unequal variance. Results which were significantly different from controls are shown with ***p ≤ 0.001, ****p ≤ 0.0001.

**Figure 2:**
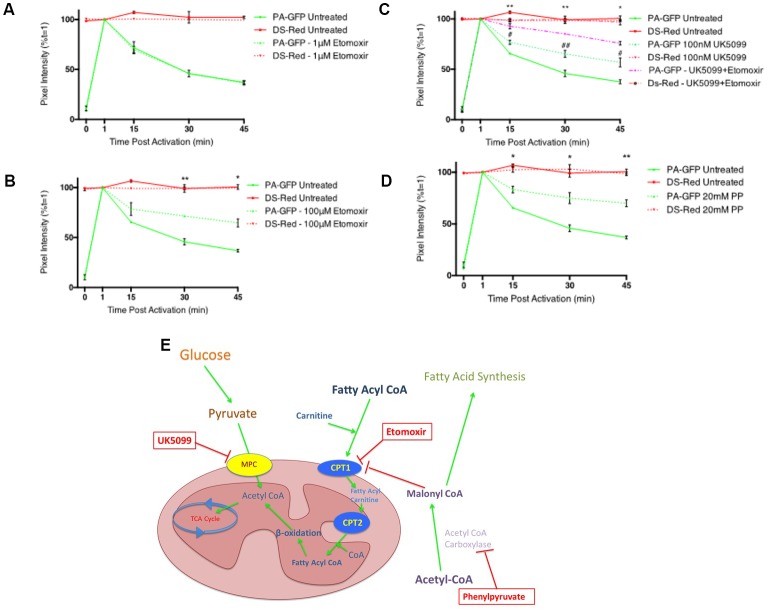
Inhibitors of fatty acid metabolism and pyruvate transport decrease mitochondrial fusion dynamics in differentiated PC12 cells. **(A,B)** Quantitative data shows inhibition of fusion by 100 μM etomoxir treated cells (dashed green line) compared to controls (bold green line) while no inhibition of fusion was observed in cells treated with 1 μM etomoxir. **(C)** Quantitative data shows synergistic inhibition of fusion in UK5099 treated cells (dashed green line) compared to controls (bold green line) and 100 μM etomoxir + UK5099 treated cells (dashed magenta line). ^#^Indicates significant differences between UK5099 treated cells and untreated cells while *indicates significant differences between UK5099 treated cells and cells treated with UK5099 + etomoxir. **(D)** Quantitative data shows inhibition of fusion in phenylpyruvate treated cells (dashed green line) compared to controls (bold green line). **(E)** Diagram of inhibitor targets. Data presented as mean ± SEM, *n* = 3. Unpaired *t*-test at each time point with Welch correction for unequal variance. Results which were significantly different from controls are shown with *p ≤ 0.05, **p ≤ 0.01, ^#^*p* ≤ 0.05, ^##^*p* ≤ 0.01.

To study the ability of fatty acid metabolism and pyruvate transport inhibitors on mitochondrial fusion dynamics a real-time confocal microscopy assay was established. Differentiated PC12 cells transfected with pDsRed2-mito and PA-GFP-mito were analyzed by confocal microscopy over a 45 min period (Figures 1A–E) and the PA-GFP-mito mean pixel intensities decreased in a time-dependent manner (Figure 1F), indicative of mitochondrial fusion. Addition of the CPT1 inhibitor, etomoxir, resulted in a dose-dependent inhibition of mitochondrial fusion rates in differentiated PC12 cells (Figures 2A,B). These results suggest a potential role for fatty acid oxidation in maintaining mitochondrial dynamics homeostasis.

Pyruvate constitutes a critical branch point in cellular carbon metabolism as it is an end product of glycolysis and a major substrate for the TCA cycle while also being involved in anabolic pathways for lipid synthesis, amino acid biosynthesis and gluconeogenesis. Transport of pyruvate into the mitochondria is performed by the MPC. Addition of the MPC inhibitor, UK5099, resulted in a significant decrease in mitochondrial fusion dynamics (Figure 2C). When UK5099 and etomoxir were pre-incubated for 1 h prior to beginning the fusion experiment, mitochondrial fusion dynamics were synergistically inhibited (Figure 2C). These data further suggest that fatty acid oxidation supplies a significant portion of acetyl-CoA for the TCA cycle, and when both pyruvate transport and fatty acid oxidation are inhibited, the cell cannot maintain mitochondrial fusion dynamics.

Phenylpyruvate has been shown to competitively inhibit acetyl-CoA carboxylase in extracts from rat brains (Land and Clark, 1973). Acetyl CoA carboxylase catalyzes the irreversible carboxylation of acetyl-CoA to produce malonyl-CoA through its two catalytic activities, biotin carboxylase (BC) and carboxyltransferase (CT; Vagelos et al., 1963). Treatment of cells with the acetyl CoA carboxylase inhibitor, phenylpyruvate, induced a significant decrease in mitochondrial fusion dynamics (Figure 2D). Acetyl CoA carboxylase catalyzes the conversion of acetyl CoA to malonyl CoA and is an important metabolic control point for fatty acid synthesis. Decreased malonyl CoA in the cell would result in an increase in CPT1 activity, and subsequently fatty acid oxidation.

### The Pyruvate Dehydrogenase Activator Dichloroacetate Restores Mitochondrial Fusion Activity, JO_**2**_ and ΔΨ_m_ in Metabolically Challenged Cells

The mitochondrial PDH complex catalyzes the oxidative decarboxylation of pyruvate to acetyl CoA, a key intermediate in the TCA cycle. When glucose supply is in excess, the combination of mitochondrial acetyl-CoA with oxaloacetate, *via* citrate formation and efflux, provides a precursor for malonyl CoA production and so limits the oxidation of fatty acids. PDH is subject to a continuous phosphorylation (inactivation)—dephosphorylation (activation) cycle catalyzed by PDHK, dedicated regulatory enzymes which phosphorylate and inactivate PDH, and PDH phosphatases (PDHPs) which dephosphorylate and activate PDH (Holness and Sugden, 2003). Dichloroacetate is an inhibitor of PDHK, and thus activates PDH promoting the conversion of pyruvate to acetyl-CoA and thus encouraging glucose use as a primary energy source. We investigated if increasing flux through PDH could overcome the deficits in mitochondrial fusion dynamics seen with the metabolic inhibitors used in Figure 2E.

**Figure 3:**
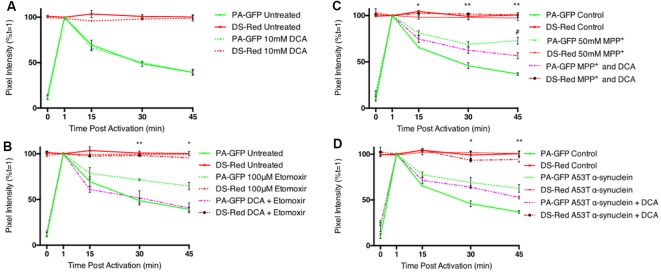
Dichloroacetate restores mitochondrial fusion dynamics in etomoxir treated PC12 cells. Differentiated PC12 cells were pre-incubated with 10 mM DCA and the effects on mitochondrial fusion dynamics assayed by confocal microscopy. **(A)** Quantitative data shows no inhibition of fusion in DCA treated cells (dashed green line) compared to controls (bold green line). **(B)** Quantitative data shows the inhibition of fusion in etomoxir treated cells was reversed by DCA. **(C)** Quantitative data shows the inhibition of fusion in MPP^+^ treated cells was partially reversed by DCA. ^#^Indicates significant differences between MPP^+^ treated cells and cells which have been treated with both MPP^+^ and DCA. **(D)** Overexpression of mutant (A53T) α-synuclein decreased mitochondrial fusion rates in differentiated PC12 cells. This effect was not reversed by a 1 h incubation with 10 mM DCA. Data presented as mean ± SEM, *n* = 3 (*n* = 4 for MPP^+^ and DCA). Unpaired *t*-test at each time point with Welch correction for unequal variance. Results which were significantly different from controls are shown with ^*,#^*p* ≤ 0.05, **p ≤ 0.01.

Addition of DCA had no effect on mitochondrial fusion dynamics (Figure 3A), suggesting that the flux of pyruvate transport into the cell and mitochondrion is sufficient to ensure maximum levels of mitochondrial fusion. Inhibition of mitochondrial fusion dynamics with the CPT 1 inhibitor, etomoxir, was reversed by co-incubation with DCA (Figure 3B). The parkinsonian-inducing neurotoxin, MPP^+^, decreased mitochondrial fusion dynamics in differentiated cells, however, this effect was found to be partially reversed with DCA (Figure 3C). Mutations in α-synuclein are known to result in familial PD and this protein accumulates into insoluble protein aggregates known as Lewy bodies. Overexpression of a mutant form of this protein (A53T) resulted in a significant decrease in mitochondrial fusion dynamics, however, this effect was not reversed with a 1 h incubation with DCA (Figure 3D).

An increased pyruvate flux into the mitochondrion, and subsequent generation of increased acetyl CoA is sufficient to overcome the effect of inhibition of fatty acid oxidation on mitochondrial fusion dynamics. DCA also shows some effectiveness at overcoming the toxic effects of high concentrations of MPP^+^, likely through increased FADH_2_-linked respiration through complex II. However, in the time-frame of these experiments, DCA cannot overcome fusion deficits seen upon protein misfolding stress, such as overexpression of mutant α-synuclein.

**Figure 4:**
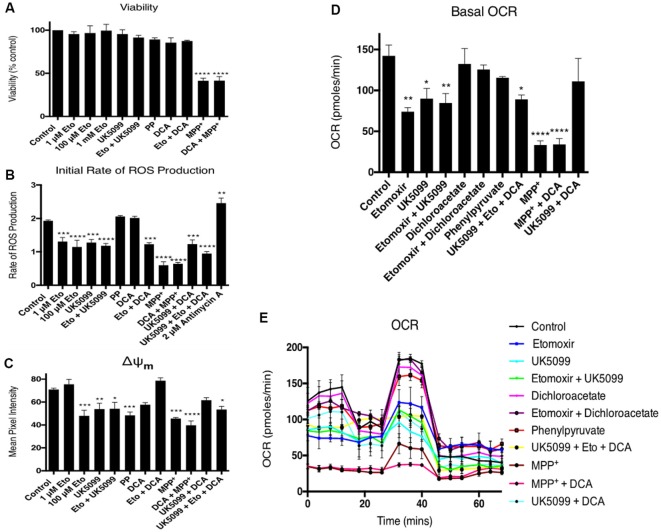
Dichloroacetate reverses etomoxir-induced effects on ΔΨ_m_ and JO_2_. Differentiated PC12 cells were pre-incubated with a range of inhibitors ± dichloroacetate and cell viability, reactive oxygen species (ROS) production, ΔΨ_m_ and JO_2_ oxygen consumption rate (OCR) assayed. **(A)** Only a 1-h incubation with 50 mM MPP^+^ significantly reduced cell viability which was not rescued by 10 mM DCA did not rescue any viability. **(B)** One-hour treatment with 1 μM etomoxir, 100 μM etomoxir, 100 nM UK5099 and 50 mM MPP^+^ all significantly reduced the initial rate of ROS production as measured by 2′,7′-dichlorodihydrofluorescein di-acetate (H_2_DCFDA) fluorescence. Co-incubation with DCA did not reverse this effect while 2 μM antimycin A treatment significantly increased the rate of ROS production. **(C)** One-hour treatment with 1 μM etomoxir, 100 μM etomoxir, 100 nM UK5099, 20 mM phenylpyruvate and 50 mM MPP^+^ all significantly reduced ΔΨ_m_ as measured by TMRM fluorescence. Co-incubation with 10 mM DCA reversed this effect in cells treated with 100 μ M etomoxir and 100 nM UK5099 but not MPP^+^. **(D,E)** One-hour treatment with 1 μM etomoxir, 100 μM etomoxir, 100 nM UK5099, and 50 mM MPP^+^ all significantly reduced the basal rate of oxygen consumption. Co-incubation with DCA reversed this effect in cells treated with 100 μ M etomoxir and 100 nM UK5099 but not MPP^+^. Data presented as mean ± SEM, *n* = 3. A one-way analysis of variance (ANOVA) was performed on each data set. Results which were significantly different from controls are shown with *p ≤ 0.05, **p ≤ 0.01, ***p ≤ 0.001 and ****p ≤ 0.0001.

Off-target effects of high etomoxir concentrations may involve inhibition of complex I and adenine nucleotide translocase activities (Divakaruni et al., 2018; Raud et al., 2018; Yao et al., 2018). To test the effects of the inhibitors on bioenergetics function during the timeframe of the fusion experiments, differentiated PC12 cells were assayed for cell viability, ROS production, mitochondrial membrane potential (ΔΨ_m_) and oxygen respiration rates (JO_2_). None of the inhibitors used had any effect on cell viability, except for a reduction caused by MPP^+^ (Figure 4A). Etomoxir decreased JO_2_, ΔΨ_m_ and ROS production rates (Figures 4B–E). Dichloroacetate reversed or partially reversed effects on these parameters except for ROS (Figures 4B–E). UK5099 treatment also decreased JO_2_, ΔΨ_m_ and ROS production rates. This reduction in JO_2_ and ΔΨ_m_ was rescued by co-incubation with DCA (Figures 4B–D). Phenylpyruvate decreased mitochondrial fusion dynamics and ΔΨ_m_, but had no effect on JO_2_ or ROS generation. MPP^+^ decreased JO_2_, ΔΨ_m_ and ROS production rates (Figures 4B–E), the effects of which were not reversed by DCA. These data suggest that the increased flux through PDH following DCA treatment allows a restoration of normal oxidative phosphorylation and ΔΨ_m_ which returns mitochondrial fusion dynamics to their normal state.

## Discussion

Limited research has been carried out into the effect of metabolic substrate transport or nutrient availability on mitochondrial dynamics, except in cases of starvation or high glucose treatment. This is somewhat surprising considering fatty acid oxidation and amino acid metabolism can significantly contribute to cellular metabolism in most tissues of the body. In particular the brain is thought to use glucose, and lactate from neighboring glial cells, almost exclusively as a substrate for ATP production but recent research has suggested that certain subsets of neurons may use fatty acid oxidation as a nutrient source under certain conditions (Panov et al., 2014; Schulz et al., 2015). The neuron-specific form of CPT1 (CPT1c) has been shown to have negligible catalytic activity in comparison to other isoforms and plays a role in hypothalamic control of energy homeostasis, hippocampal-dependent spatial learning and trafficking of AMPA receptor subunits to the neuronal plasma membrane (Wolfgang et al., 2006; Carrasco et al., 2013).

This study found that the CPT1 inhibitor etomoxir resulted in a significant reduction in mitochondrial fusion dynamics in differentiated PC12 cells. The data suggests differentiated PC12 cells use the β-oxidation of fatty acids as an energy source to drive mitochondrial fusion. Indeed, recent research by Panov et al. (2014) sheds more light on the ability of neurons to use fatty acids as an energy substrate. This group isolated synaptic mitochondria from rats and found that incubation with palmitoyl carnitine alone resulted in a higher level of state 4 respiration than incubation with glutamate or pyruvate and that palmitoyl carnitine also showed significant state 3 respiration rates although not as high as glutamate or pyruvate. In agreement with this (Stoll et al., 2015) showed that neural stem/progenitor cells (NSPCs) demonstrate sustained decreases in oxygen consumption upon treatment with etomoxir. This suggests evidence that neurons use a variety of energy sources, most likely due to their high energy demand, and inhibition of one of these, such as fatty acid oxidation could have a significant effect on neuronal respiration rates and subsequently, mitochondrial fusion dynamics.

Even accounting for β-oxidation of fatty acids as an energy source for differentiated PC12 cells, it seems unlikely that it contributes enough to account for the ~70% reduction in fusion dynamics observed with the 1 mM etomoxir treatment (Data not shown). Pike et al. (2011) showed that etomoxir reduced cellular ATP levels, NADPH levels and increased intracellular ROS in glioblastoma SF188 cells while (Merrill et al., 2002) found that etomoxir induced oxidative stress, reduced mitochondrial membrane potential and ATP levels and increased superoxide generation. In PC12 cells, the etomoxir-induced decrease in fusion dynamics correlates with a decrease in JO_2_, ΔΨ_m_ and ROS production (Figures 4B–D). Assessing these results, it is likely that treatment with etomoxir has a cumulative effect, whereby the inhibition of fatty acid metabolism may play a role in the reduction in fusion rates and this is exacerbated by the toxic effects of etomoxir, such as the decrease in mitochondrial membrane potential which can decrease the likelihood of a fusion event occurring (Twig et al., 2008).

A number of α-cyanocinnamate analogs, such as UK5099, have been identified as specific and potent inhibitors of carrier activity (Halestrap, 1975). Treatment with UK5099 resulted in a significant inhibition of mitochondrial fusion dynamics in differentiated PC12 cells (Figure 2C). Interestingly, there was approximately a 30% inhibition of fusion observed after UK5099 treatment, which is less than one would expect by completely blocking pyruvate transport into the mitochondrion as it is a major substrate of the TCA cycle. One reason for the low level of fusion inhibition observed is possibly due to the passive diffusion of pyruvate across the mitochondrial membrane that is not inhibited by UK5099 and provides another means of entry into the TCA cycle (Hildyard et al., 2005). In PC12 cells the UK5099-induced decrease in fusion dynamics correlates with a decrease in JO_2_, ΔΨ_m_ and ROS production (Figures 4B–D). Co-incubation with UK5099 and etomoxir resulted in cumulative effect of fusion inhibition with around 60% overall inhibition (Figure 2C). This result again suggests that β-oxidation of fatty acids may have a more significant input into PC12 cell metabolism than is currently appreciated and certainly plays a role in the maintenance of mitochondrial fusion-fission dynamics.

By incubating PC12 cells with phenylpyruvate, a decrease in malonyl CoA levels and a subsequent increase in flux through the fatty acid β-oxidation pathway, leading to an increase in mitochondrial fusion rates, might be expected. However, a significant decrease was observed (Figure 2D). This result was not entirely surprising as phenylpyruvate has a wide range of substrates involved in a variety of metabolic pathways, which may have impacted ATP production and/or the ability of the mitochondria to fuse. In PC12 cells the phenylpyruvate-induced decrease in fusion dynamics correlates with a slight decrease in ΔΨ_m_ but no effect on cell viability, JO_2_ or ROS production (Figures 4A–E). This indicates that the phenylpyruvate-induced decrease in fusion dynamics is ΔΨ_m_ dependent.

Phenylketonuria is an inherited disorder resulting in decreased metabolism of the amino acid phenylalanine as a result of a mutation in the gene for the hepatic enzyme phenylalanine hydroxylase (PAH), rendering it non-functional. The resultant accumulation of phenylalanine is converted into phenylpyruvate (Christ, 2003). Phenylketonuria presents with symptoms affecting the brain including microcephaly, hyper-activity and severe learning disabilities (Al Hafid and Christodoulou, 2015). The disruption of mitochondrial dynamics by phenylpyruvate supports the findings that some of the symptoms seen in phenylketonuria along with the described reduction in myelination are related to increased phenylpyruvate concentrations (Land and Clark, 1973).

As seen in Figure 3A, treatment with 10 mM DCA has no effect on mitochondrial fusion rates, suggesting that pyruvate utilization is already sufficient to drive normal mitochondrial fusion dynamics. Due to other toxic side effects of etomoxir, it is possible that the decrease in fusion dynamics observed with 100 μM treatment is due to a combination of decreased fatty acid oxidation inhibition with decreased mitochondrial bioenergetic function. The results in Figures 4A–D show that etomoxir induces a decrease in ΔΨ_m_ and JO_2_, and also in ROS generation. This suggests that etomoxir is decreasing fusion dynamics as a consequence of CPT inhibition and subsequent inhibition of ΔΨ_m_ and JO_2_, rather than any effect on the ETC complexes that would more likely result in increased ROS production. The etomoxir-induced decrease in ROS generation may be due to decreased flux through PDH, a known generator of superoxide in brain mitochondria (Quinlan et al., 2014). Treatment with DCA overcomes the decrease in fusion dynamics seen with 100 μM etomoxir treatment and returns fusion rates to control levels (Figure 3B), as well as reversing the effects on ΔΨ_m_ and JO_2_ (Figures 4C,D). This result suggests that the inhibition of fusion following etomoxir treatment was due to a decrease in fatty acid oxidation and subsequent reduction in mitochondrial acetyl CoA levels and that increasing pyruvate decarboxylation by PDH compensates for loss of fatty acid oxidation.

Many patients with defects in long chain fatty acid oxidation present with neurological symptoms (Kompare and Rizzo, 2008) and altered lipid metabolism has also been identified in patients with a number of neurodegenerative disorders (Adibhatla and Hatcher, 2008). These results suggest that increasing metabolic flux through PDH and stimulating a maintenance of mitochondrial fusion dynamics and general cellular homeostasis could be a potential therapeutic for patients with defective lipid metabolism.

Decreases in mitochondrial electron transport chain activities are implicated in the pathogenesis of neurodegenerative disorders (Pathak and Davey, 2008) and the presence of energy thresholds in brain mitochondria (Kilbride et al., 2008, 2011; Telford et al., 2009) are neurotherapeutic targets (Telford et al., 2010). In this study, the decrease in mitochondrial fusion dynamics caused by the parkinsonian neurotoxin, MPP^+^, was partially reversed by DCA (Figure 3C). However, the extensive decreases in cell viability, ROS generation and ΔΨ_m_ (Figures 4A–C) induced by MPP^+^ were not reversed by DCA. MPP^+^ accumulates into mitochondria *via* Nernstian transport kinetics (Davey et al., 1992) and is a complex I targeted neurotoxin. The partial restoration of mitochondrial dynamics by DCA may result from increased metabolic flux through PDH, thus generating TCA intermediates that facilitate FADH_2_-linked respiration.

Misfolded α-synuclein has also been shown to decrease complex I activity (Chinta et al., 2010) and the brains of PD patients exhibit reduced complex I activity (Devi et al., 2008). Indeed, overexpression of mutant (A53T) α-synuclein reduced mitochondrial fusion dynamics in differentiated PC12 cells, however, this effect was not reversed by short-term DCA treatment (Figure 3D). Aggregated α-synuclein may interfere with other components of the fusion machinery and prevent any changes in fusion dynamics or the overall damaging effect of aggregated α-synuclein may be too great for a short-term treatment with DCA to overcome. Further investigation is required to explore if altering pyruvate flux may provide potential for treatment in the pathogenesis of a neurodegenerative disorder, such as PD.

DCA treatment abrogated the inhibitory effects of UK5099 on ΔΨ_m_ and JO_2_ (Figures 4C–E). This result is somewhat surprising as one would expect blocking pyruvate transport into the mitochondria would reduce any substrate for activated PDH to work on. However, passive transport of pyruvate may be sufficient to provide enough substrate for activated PDH to maintain ΔΨ_m_ and JO_2_ even in the presence of reduced mitochondrial pyruvate concentrations.

These results show that regulating metabolic flux in the cell can affect mitochondrial fusion dynamics in differentiated PC12 cells. Importantly, fatty acid oxidation may play a larger role in the maintenance of mitochondrial fusion in this cell type than previously anticipated. The inhibitory effects on fusion dynamics seen are mediated through alterations in ΔΨ_m_ and JO_2_ while ROS generation appears to have no significant input into this process.

## Data Availability

The raw data supporting the conclusions of this manuscript will be made available by the authors, without undue reservation, to any qualified researcher.

## Author Contributions

DO’H performed the experiments and co-wrote the manuscript. NA and GMD performed the fusion experiments in syn PC12 cells. JH co-designed the experiments. GPD designed, oversaw the study, and co-wrote the manuscript.

## Conflict of Interest Statement

The authors declare that the research was conducted in the absence of any commercial or financial relationships that could be construed as a potential conflict of interest.
